# Technical Performance Evaluation of the MyT4 Point of Care Technology for CD4+ T Cell Enumeration

**DOI:** 10.1371/journal.pone.0107410

**Published:** 2014-09-17

**Authors:** Matilu Mwau, Silvia Kadima, Joy Mwende, Maureen Adhiambo, Catherine Akinyi, Marta Prescott, Judi Lusike, Jackson Hungu, Lara Vojnov

**Affiliations:** 1 Kenya Medical Research Institute, Nairobi, Kenya; 2 Clinton Health Access Initiative, Nairobi, Kenya; CEA, France

## Abstract

**Objective:**

Though absolute CD4+ T cell enumeration is the primary gateway to antiretroviral therapy initiation for HIV-positive patients in all developing countries, patient access to this critical diagnostic test is relatively poor. We technically evaluated the performance of a newly developed point-of-care CD4+ T cell technology, the MyT4, compared with conventional CD4+ T cell testing technologies.

**Design:**

Over 250 HIV-positive patients were consecutively enrolled and their blood tested on the MyT4, BD FACSCalibur, and BD FACSCount.

**Results:**

Compared with the BD FACSCount, the MyT4 had an r^2^ of 0.7269 and a mean bias of −23.37 cells/µl. Compared with the BD FACSCalibur, the MyT4 had an r^2^ of 0.5825 and a mean bias of −46.58 cells/µl. Kenya currently uses a CD4+ T cell test threshold of 350 cells/µl to determine patient eligibility for antiretroviral therapy. At this threshold, the MyT4 had a sensitivity of 95.3% (95% CI: 88.4–98.7%) and a specificity of 87.9% (95% CI: 82.3–92.3%) compared with the BD FACSCount and sensitivity and specificity of 88.2% (95% CI: 79.4–94.2%) and 84.2% (95% CI: 78.2–89.2%), respectively, compared with the BD FACSCalibur. Finally, the MyT4 had a coefficient of variation of 12.80% compared with 14.03% for the BD FACSCalibur.

**Conclusions:**

We conclude that the MyT4 performed well at the current 350 cells/µl ART initiation eligibility threshold when used by lower cadres of health care facility staff in rural clinics compared to conventional CD4+ T cell technologies.

## Introduction

CD4+ T cell enumeration is critical to determine antiretroviral therapy (ART) eligibility in all high HIV burden developing countries [1]. The lack of available funding prevents treating all HIV diagnosed individuals; therefore, CD4+ T cell testing identifies the sickest patients in most need of ART. Additionally, CD4+ T cell enumeration is the most predictable indicator of HIV disease progression [2]–[5] and is more reliable for determining ART eligibility than symptomatic staging [6]–[8].

The WHO now recommends using an ART eligibility threshold of 500 cells/µl for HIV-positive individuals over five years of age [1]. In 2011, UNAIDS set a target to provide ART to 15 million people by 2015 [9]. Though strongly recommended by the WHO, there is significant lack of access to CD4+ T cell testing throughout Africa. It is estimated that only 45% of HIV-positive individuals in several high HIV burden countries have access to on-site CD4+ T cell testing. Approximately 9 million individuals are on ART of the almost 28 million who are now eligible according to the 2013 WHO guideline recommendations [1]. Increasing CD4+ T cell testing through decentralization may provide increased access to this critical gateway to ART.

CD4+ T cell testing is primarily available at centralized laboratories with conventional laboratory-based CD4+ T cell testing technologies. This system is insufficient to provide the full CD4+ T cell testing need. Patients who do not have reliable on-site access to such testing generally have to make multiple visits to the health care facility as samples are referred to the laboratory. Often patients are lost to follow up waiting for test results due to long test turnaround times [10], [11]. Unfortunately, the cost of building new conventional laboratories is prohibitive in most countries, while finding additional trained technical staff is difficult.

Point-of-Care diagnostic technologies offer an opportunity to alleviate such critical testing needs in settings lacking on-site testing. POC technologies generally do not require consistent electricity, are easy to use, capable of providing test results within minutes, and can withstand hot and humid conditions [12]. POC CD4+ T cell enumeration technologies have previously shown good correlation with conventional technologies [13]–[15]. Additionally, POC CD4+ T cell testing reduced patient loss to follow-up and the test turnaround time, while increasing the number of patients who initiated ART compared to the conventional, referral testing system [10], [11].

The MyT4 POC CD4+ T cell enumeration technology has been developed and has the potential to help fill the current CD4+ T cell testing gaps. We, therefore, sought to evaluate the technical field performance of the technology compared with two common conventional CD4+ T cell technologies, the BD FACSCalibur and BD FACSCount. In this study, the MyT4 was placed in the Comprehensive Care Clinic of two health care facilities in rural Kenya and operated by health care facility staff. The test results were compared to those from conventional CD4+ T cell technologies for accuracy and repeatability.

## Materials and Methods

### Study Population

Participants were recruited in January and February 2014 at the Comprehensive Care Clinic of two health care facilities in the Busia County of Western Province, Kenya: Alupe Sub-District Hospital and Nambale Health Center. All HIV-positive patients over 18 years of age attending the selected health care facilities for treatment and care were eligible for inclusion in this study. Only patients who provided written informed consent prior to testing were enrolled in the study. All patient visits and capillary health care facility MyT4 testing occurred during regular clinic hours between 9am and 3pm with health care facility temperatures ranging between 20°C to 30°C. This study was reviewed and approved by the Kenya Medical Research Institute Ethical Review Committee (Protocol No. 2657) and was conducted in accordance with the ethical standards of the Helsinki Declaration of 1975, as revised in 2000.

### Study Design

This independent technical methods comparison study compared the performance of the MyT4 POC CD4+ T cell technology (developer and manufacturer: Zyomyx, Inc. Fremont, CA, USA; distributor: Mylan Laboratories, Ltd., Canonsburg, PA, USA) using fresh, finger-prick capillary samples collected and tested by health care facility staff with conventional CD4+ T cell testing of the BD FACSCalibur and BD FACSCount (Becton Dickinson, East Rutherford, NJ, USA) performed by trained laboratory technicians using matched EDTA blood. Informed consenting patients were enrolled consecutively before providing a finger-prick blood sample and a venipuncture EDTA blood sample. Qualified and trained health care facility staff, comprised of both nurses and laboratory technicians, performed the MyT4 test and drew venipuncture EDTA blood for conventional laboratory CD4+ T cell testing. Demographic data and test results from each patient was collected and entered into a Microsoft Excel database. All CD4+ T cell testing, including both conventional CD4+ T cell tests, were processed on the same day as sample extraction.

Health care facility staff performed the MyT4 test in the clinic using the capillary finger-prick samples. Nurses performed the majority of the MyT4 testing. After the operator pricked the patient's finger, a provided blood capillary tube collected 100 µl of blood. The capillary tube was then inserted into the provided cartridge and blood dispensed into the lid using a provided plunger. Within no more than a few minutes of blood dispensing, the cartridge was placed into the mixer of the device for approximately four minutes before being placed into the spinner of the device for a further four minutes. The lid of the spinner must be manually twisted prior to spin commencement to break the cartridge membrane. Finally, after spin completion the cartridge is inserted into the simplified microscope in the device to read three internal controls and the CD4+ T cell test result. Reading must be performed within five minutes of test completion. The EDTA blood sample from each patient was delivered to the Kenya Medical Research Institute (KEMRI) laboratory for testing using the BD FACSCalibur and to the Alupe Sub-District Hospital laboratory for testing using the BD FACSCount. EDTA blood was used for testing the repeatability of the MyT4 test in the KEMRI laboratory. The laboratory operators were blinded of the MyT4 results, while the MyT4 operators were blinded of the conventional test results. CD4+ T cell testing using both the MyT4 and conventional technologies were performed according to manufacturers' instructions, by trained staff.

For clinical management, patients were only provided with the CD4+ T cell result from the conventional CD4+ T cell technologies. Conventional CD4+ T cell technologies are enrolled in External Quality Assurances (EQA) schemes, such as the Western Province External Quality Assurance Scheme (WEPEQAS) and CDC Inter-Laboratory EQA. All laboratory technologists performing the conventional CD4+ T cell technologies included in this study are trained annually in good laboratory practice, immunophenotyping for flow cytometry, and biosafety. Daily controls were run for each technology.

### Statistical Analysis Methods

The technical performance characteristics of the MyT4 were analyzed using standard statistical methods for evaluating diagnostic technologies. Bland-Altman analyses were performed to determine the bias and 95% limits of agreement between the two selected technologies. The y-axis on each Bland-Altman plot uses the difference between the two methods (test technology – gold standard). The absolute CD4+ T cell counts from the MyT4 were directly compared to those from both the BD FACSCount and BD FACSCalibur conventional CD4+ T cell technologies using linear regression analysis and calculating the coefficient of determination (r^2^). Repeatability was calculated on paired samples in the laboratory on the same instrument by the same technician for both the MyT4 and BD FACSCalibur and determined by the coefficient of variation. Finally, the sensitivity, specificity and misclassification of the MyT4 were calculated compared with the conventional CD4+ T cell technologies using the following thresholds: 100 cells/µl, used for Cryptococcal reflex testing; 350 cells/µl, the current ART initiation eligibility threshold; and 500 cells/µl, the 2013 WHO recommended ART initiation eligibility threshold. Misclassification was defined using the below equations:

Upward misclassification percentage: # of patients incorrectly identified as above the threshold using the MyT4/# of patients identified as below the threshold using the conventional CD4+ T cell technology

Downward misclassification percentage: # of patients incorrectly identified as below the threshold using the MyT4/# of patients identified as above the threshold using the conventional CD4+ T cell technology.

All statistical analyses were performed with GraphPad Prism, STATA and/or Microsoft Excel.

## Results

**Figure 1:**
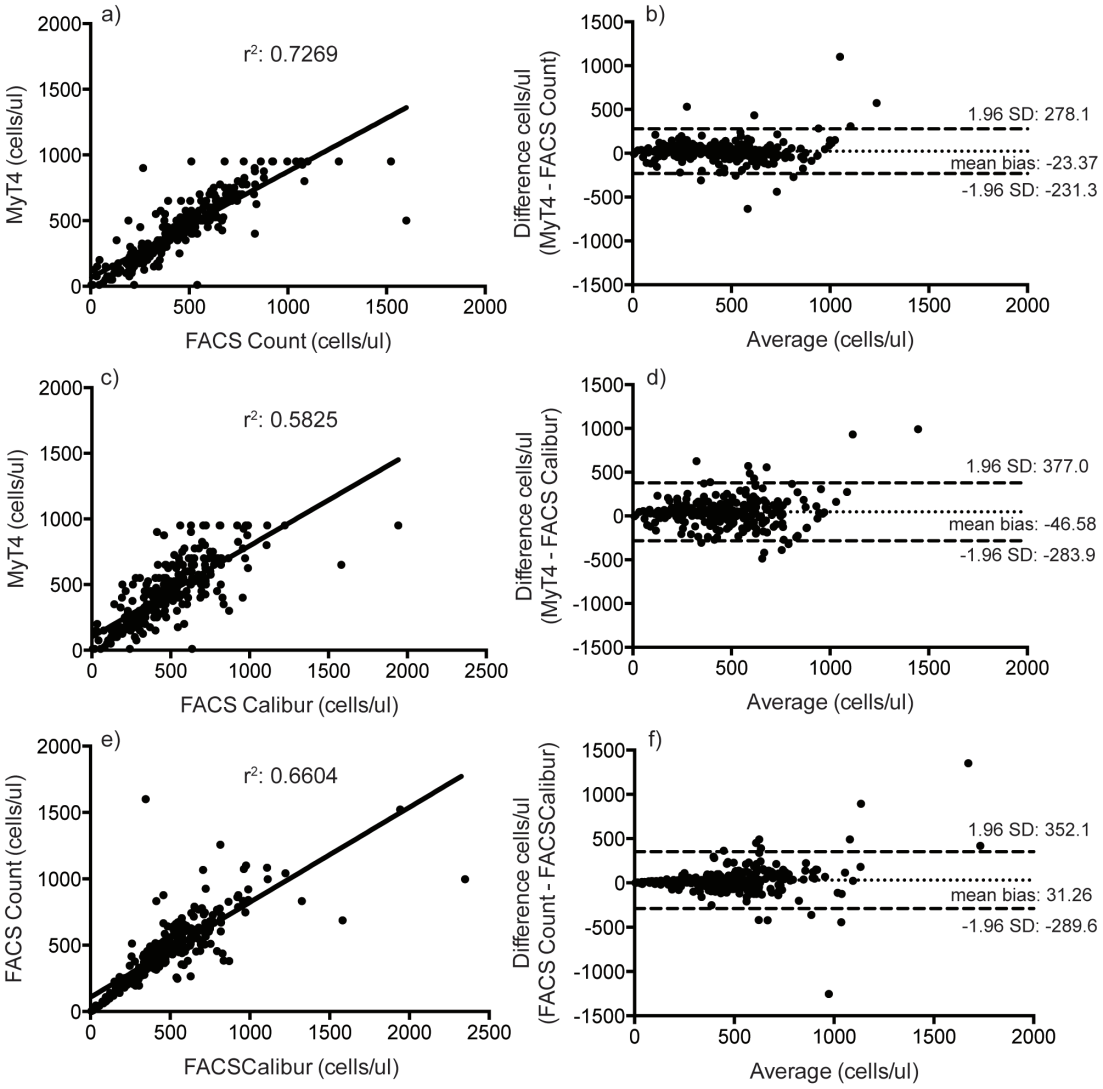
Linear regression (a, c, e) and Bland-Altman (b, d, f) analyses of absolute CD4+ T cell counts between the MyT4 and BD FACSCount (a and b); the MyT4 and BD FACSCalibur (c and d); and BD FACSCount and BD FACSCalibur (e and f).

Two hundred and seventy-six patients were consecutively enrolled and included in the study analysis with the number of test results above and below the three tested CD4+ T cell thresholds highlighted in Table 1. Approximately 70% of the participants were female. The majority of patients (86.5%) were between 26 and 55 years of age with the mean age at enrollment being 42 years. To understand the technical performance of the MyT4, we compared it to two well-accepted conventional CD4+ T cell technologies: the BD FACSCalibur and BD FACSCount. The MyT4 has a CD4+ T cell range from 10–950 cells/µl. We first compared the MyT4 with the BD FACSCount from 267 enrolled HIV-positive individuals. Between the two technologies, the coefficient of determination, r^2^, was 0.7269 and the mean bias was −23.37 cells/µl (95% LOA: -231.3–278.1 cells/µl) (Figure 1a and b).

**Table 1:** Number of CD4+ T cell test results by technology and CD4+ T cell threshold used.

		276 total patients enrolled		
		FACSCalibur	FACSCount	MyT4
	Total CD4 results per technology	270	267	272
Number of CD4 results below or above specified threshold	Below 100 cells/µl	12	14	18
	Above 100 cells/µl	258	253	254
	Below 350 cells/µl	85	85	106
	Above 350 cells/µl	185	182	166
	Below 500 cells/µl	154	161	178
	Above 500 cells/µl	116	106	94

We next compared the MyT4 with the BD FACSCalibur from 270 enrolled HIV-positive individuals. Between the two technologies, the coefficient of determination, r^2^, was 0.5825 and the mean bias was −46.58 cells/µl (95% LOA: −283.9–377.0 cells/µl) (Figure 1c and d). Since we tested each patient's sample using all three technologies, the MyT4, BD FACSCalibur and BD FACSCount, we also compared the performance between the BD FACSCalibur and BD FACSCount. Between the two technologies, the coefficient of determination, r^2^, was 0.6604 and the mean bias was −31.26 (95% LOA: −289.6–352.1 cells/µl) (Figure 1e and f).

The MyT4 produced CD4+ T cell results with a slightly lower absolute count than both of the conventional CD4+ T cell technologies. Both of the conventional CD4+ T cell technologies identified approximately 31% of all patients included in the study as having a CD4+ T cell count below the currently used ART initiation eligibility threshold in Kenya of 350 cells/µl. The MyT4 identified approximately 39% of all patients as having a CD4+ T cell count below the currently used ART initiation eligibility threshold of 350 cells/µl.

Using the current ART initiation eligibility threshold of 350 cells/µl, the MyT4 had a sensitivity of 95.3% (95% CI: 88.4–98.7%) and specificity of 87.9% (95% CI: 82.3–92.3%) to correctly identify ART initiation eligibility when compared with the BD FACSCount (Table 2). Compared with the BD FACSCalibur at the same CD4+ T cell threshold, the sensitivity and specificity of the MyT4 were 88.2% (95% CI: 79.4–94.2%) and 84.2% (95% CI: 78.2–89.2%), respectively (Table 3).

**Table 2:** Sensitivity, specificity, upward and downward misclassification rates, and positive and negative predictive values of the MyT4 CD4+ T cell technology compared with the BD FACSCount across three CD4+ T cell thresholds.

		Sensitivity (95% CI)	Specificity (95% CI)	Upward misclassification	Downward misclassification	Total misclassification	Positive Predictive Value	Negative Predictive Value
**CD4+ T cell threshold**	100 cells/µl	71.4% (41.9–91.6%)	96.8% (93.9–98.6%)	28.6%	3.2%	4.5%	55.6%	98.4%
	350 cells/µl	95.3% (88.4–98.7%)	87.9% (82.3–92.3%)	4.7%	12.1%	9.7%	78.6%	97.6%
	500 cells/µl	91.9% (86.6–95.6%)	75.5% (66.2–83.3%)	8.1%	24.5%	14.6%	85.1%	86.0%

**Table 3:** Sensitivity, specificity, upward and downward misclassification rates, and positive and negative predictive values of the MyT4 CD4+ T cell technology compared with the BD FACSCalibur across three CD4+ T cell thresholds.

		Sensitivity (95% CI)	Specificity (95% CI)	Upward misclassification	Downward misclassification	Total misclassification	Positive Predictive Value	Negative Predictive Value
**CD4+ T cell threshold**	100 cells/µl	66.7% (34.9–90.1%)	96.1% (93.0–98.1%)	33.3%	3.9%	5.2%	44.4%	98.4%
	350 cells/µl	88.2% (79.4–94.2%)	84.2% (78.2–89.2%)	11.8%	15.8%	14.4%	72.1%	93.9%
	500 cells/µl	89.0% (82.9–93.4%)	66.1% (56.7–74.7%)	11.0%	33.9%	20.7%	77.8%	81.7%

Most importantly, we wanted to understand the programmatic implications of using the MyT4 in the intended field settings. We, therefore, also analyzed the performance of the MyT4 to correctly identify patients as above or below several specific CD4+ T cell thresholds compared with both conventional CD4+ T cell technologies. Upward misclassification rates were calculated as the number of patients incorrectly identified as above the given threshold by the MyT4 over the number of patients identified as below the given threshold by the conventional CD4+ T cell technology. Downward misclassification rates were calculated as the number of patients incorrectly identified as below the given threshold by the MyT4 over the number of patients identified as above the given threshold by the conventional CD4+ T cell technology. Using these definitions, the MyT4 had upward and downward misclassification rates of 4.7% and 12.1%, respectively, when compared with the BD FACSCount using a 350 cells/µl threshold, resulting in 9.7% total misclassification. Misclassification rates at different CD4+ T cell thresholds compared with both the BD FACSCount and BD FACSCalibur are shown in Tables 2 and 3, respectively.

We next analyzed the ability of the MyT4 to produce similar results by performing two independent tests using the same device by the same operator in the laboratory from approximately 100 patients. This analysis produced a coefficient of variation of the MyT4 of 12.80%. The same patient samples were also repeat tested using the BD FACSCalibur and resulted in a coefficient of variation of 14.03%.

Finally, the two conventional CD4+ T cell technologies at the 350 cells/µl CD4+ T cell threshold produced a sensitivity and specificity of 89.3% (95% LOA: 80.6–95.0%) and 94.1% (95% LOA: 89.6–97.0%), respectively (Table 4). This resulted in upward and downward misclassification rates of 10.7% and 5.9%, respectively, at the 350 cells/µl ART initiation eligibility threshold (data not shown).

**Table 4:** Sensitivity and specificity of the BD FACSCount compared with the BD FACSCalibur across three CD4+ T cell thresholds.

		Sensitivity (95% CI)	Specificity (95% CI)
**CD4+ T cell threshold**	100 cells/µl	91.7% (61.5–99.8%)	98.8% (96.6–99.8%)
	350 cells/µl	89.3% (80.6–95.0%)	94.1% (89.6–97.0%)
	500 cells/µl	89.0% (82.9–93.4%)	77.4% (68.7–84.7%)

## Discussion

In Kenya there are almost 600,000 HIV-positive adults and 70,000 children on ART that require monitoring by viral load or CD4+ T cell enumeration if the former is unavailable. Additionally, over 221,000 HIV-positive adults and 70,000 children remain in pre-ART care. Current guidelines in Kenya recommend that all pre-ART patients receive two CD4+ T cell tests per year to determine ART eligibility. Furthermore, there are almost 10,000 health care facilities in Kenya and 2,000 that provide ART services to HIV-positive individuals; however, only 11% of health care facilities that provide ART services have access to on-site CD4+ T cell testing. Even if no financial constraints existed, many health care facilities in Kenya and throughout sub-Saharan Africa lack the ability to operate and manage conventional CD4+ T cell testing technologies.

Creating a decentralized diagnostic network that allows for self-sufficient health care facilities will increase patient access to critical clinical services [16]. As countries decentralize diagnostic testing, the MyT4 POC CD4+ T cell technology could be used in such health care facilities. Each test using the MyT4 technology takes approximately 12–15 minutes, including sample collection, test reading and interpretation. The device does not require constant electricity and could be used for days before recharging. Additionally, the reagents or test cartridges do not require refrigeration. The current device configuration, however, does not allow for remote data management to a central server and requires manual result and internal quality control interpretation for each test performed. Additionally, when introducing and deploying any POC CD4+ T cell technology, it is important to consider the service and maintenance, supply chain and quality assurance requirements of each technology.

Though the MyT4 technology was somewhat semi-manual requiring more than five steps to complete one test, each was relatively straightforward and could be performed by lower cadres of health care facility staff. Tests could be performed using both finger-prick capillary and venipuncture blood. Furthermore, we found that nurses performed the MyT4 using capillary finger-prick blood in the intended health care facility setting, primary clinics, comparably to the results of the conventional CD4+ T cell technologies. Further refinements of the MyT4 technology to reduce the number of steps while maintaining the technical performance standards would be ideal.

Two MyT4 devices were placed at each health care facility allowing both to manage the daily patient volumes. The technology had a relatively high maximum daily throughput of over 20 tests per six-hour health care facility working day. Because the technology required a number of steps performed by the operator throughout each test, which limited the amount of potential walk-away time, implementation would likely be most effective by designating a dedicated full-time operator. It would be difficult for a clinician or nurse to both attend to patients and operate the device throughout the day at medium or high volume health care facilities.

Throughout this technical field evaluation, error rates remained relatively low with a study-wide error rate of 9.56%. Of those only 20% were known user errors. As testing proceeded and the end-users became more comfortable with the POC CD4+ T cell technology, daily error rates fell below 5%.

The MyT4 technology requires visual quantitative interpretation of CD4+ T cell results using increments of 25 cells/µl. If a CD4+ T cell result appears to fall between two such enumeration lines, the operator should read the lower number as the CD4+ T cell count. The implications of this quantitation method are unclear; however, we observed good technical performance of this technology compared to both conventional CD4+ T cell technologies tested.

We found that the MyT4 performed well in the field by all tested metrics, including accuracy and reproducibility, compared to both conventional CD4+ T cell technologies. Compared with the BD FACSCount, the mean bias was −23.37 cells/µl while the coefficient of determination, r^2^, was 0.7269. Additionally, the MyT4 produced a coefficient of variation of 12.80%, while the BD FACSCalibur had a coefficient of variation of 14.03%. As a subset of patients received an additional MyT4 test in the laboratory, we found similar comparable results between the BD FACSCalibur and venous laboratory-tested MyT4 as well as between the venous laboratory-tested MyT4 test results and the capillary health care facility-tested MyT4 test results (data not shown). Finally, though the MyT4 has a dynamic range of 10–950 cells/µl, we found similar comparable results when analyzing only those patient samples within that dynamic range as identified by the BD FACSCalibur.

Interestingly, the performance of the MyT4 was better compared with the BD FACSCount than the BD FACSCalibur. The mean bias of the MyT4 was −23.37 cells/µl compared with the BD FACSCount, while the mean bias was −46.58 cells/µl compared with the BD FACSCalibur. Similar disparate findings have been seen previously [17]. These may be because analysis of the BD FACSCalibur can be subjective when manual gating of the CD4+ T cell population is employed. Additionally, as the repeatability analysis highlighted, relative inherent variability exists when enumerating CD4+ T cells, both due to biological and technological factors, even using the same technology, device, and operator. It is not unsurprising then that technologies with different test chemistries would not precisely compare. This further supports the suggestion that variability exists between the currently available conventional CD4+ T cell technologies and that this variability should be considered when analyzing the results of any new CD4+ T cell technologies. Careful conventional CD4+ T cell technology selection should be considered prior to starting any technical evaluation.

When employing diagnostic technologies, it is important to consider any programmatic implications of incorrectly diagnosing or misclassifying patients. The MyT4 had sensitivities and specificities at 85% or greater compared with both conventional CD4+ T cell technologies when analyzed with the current ART initiation eligibility threshold in Kenya of 350 cells/µl. Encouragingly, the technical performance results of the MyT4 showed minimal misclassification of patients at that same threshold with a total misclassification rate below 10%. Interestingly, the technical performance of the MyT4 was poorer at clinical thresholds of 100 cells/µl and 500 cells/µl and, therefore, such performance should be considered before using the MyT4 for reflex Cryptococcal testing and higher ART initiation thresholds. Based on this technical field evaluation in rural Kenya, we found that the MyT4 performed satisfactorily at the current 350 cells/µl ART initiation eligibility threshold when used by lower cadres of health care staff compared to conventional CD4+ T cell technologies.
